# Amino acids and proteomic acclimation of *Staphylococcus aureus *when incubated in a defined minimal medium supplemented with 5% sodium chloride

**DOI:** 10.1002/mbo3.772

**Published:** 2019-02-09

**Authors:** Mousa M. Alreshidi, R. Hugh Dunstan, Margaret M. Macdonald, Nathan D. Smith, Johan Gottfries, Tim K. Roberts

**Affiliations:** ^1^ Department of Biology, College of Science University of Hail Hail Saudi Arabia; ^2^ Metabolic Research Group, Faculty of Science School of Environmental and Life Sciences Callaghan New South Wales Australia; ^3^ Analytical and Biomolecular Research Facility (ABRF) University of Newcastle Callaghan New South Wales Australia; ^4^ Department of Chemistry Gothenburg University Gothenburg Sweden

**Keywords:** metabolic profiling, proteomics, *Staphylococcus aureus*, stress response

## Abstract

*Staphylococcus aureus* is a versatile bacterium that can adapt to survive and grow in a wide range of salt concentrations. This study investigated whether the cells could mount a response to survive a challenge of 5% NaCl in a minimal incubation medium that would not support cell replication. Cells were grown in liquid culture, washed and then incubated for 90 min at 37°C in a medium that contained only glycine and glucose as substrates in PBS plus trace elements. The control cells were compared with a treatment group which was incubated with an additional 5% NaCl. Significantly more glycine was taken up by the cells exposed to 5% NaCl compared with control cells, and both groups consumed 99% of the glucose supplied. The NaCl treated cells had significantly higher cytoplasmic levels of proline and glutamic acid as well as lower levels of alanine and methionine compared with the controls (*p* < 0.05). The levels of the two major cytoplasmic amino acids, aspartic acid and glycine, remained constant in control and treated cells. Proteomic analyses revealed that 10 proteins showed differential responses between the control and treatment groups. The reductions in proteins were primarily associated with processes of protein biosynthesis, pathogenicity, and cell adhesion. Since cell numbers remained constant during the incubation period in minimal medium, it was concluded that there was no cell division to support population growth. The results provided evidence that the cells in the minimal medium exposed to the NaCl treatment underwent in situ homeostatic changes to adjust to the new environmental conditions. It was proposed that this represented a phenotypic shift to form cells akin to small colony variants, with lower metabolic rates and lower levels of key proteins associated with pathogenicity.

## 1 INTRODUCTION

*Staphylococcus aureus *is an important pathogenic bacterium that causes a wide range of clinical symptoms from mild skin infections to severe life‐threatening infections (Lowy, 1998). This high level of pathogenicity was proposed to be due to the ability of the bacterium to tolerate harsh conditions, including changes in environmental parameters and in the human immune system (Alreshidi, Dunstan, Onyango, & Roberts, 2013; Onyango, Dunstan, & Roberts, 2008; Onyango, Dunstan, Gottfries, Eiff, & Roberts, 2012; Sanchez, Cabo, Margolles, & Herrera, 2010). Bacteria such as *S. aureus* are routinely subjected to substantial variations in the concentrations of nutrients and ions. This results in changes in the external osmotic pressure that demand responses in the physical and chemical structure of the bacterial cell (Graham & Wilkinson, 1992; Petersson, Kamme, & Miorner, 1999) and therefore a number of mechanisms have evolved in bacteria to survive osmotic changes and ensure survival.

*Staphylococcus aureus* is well known for its adaptability to survive in a variety of salt concentrations (Alreshidi et al., 2016; Romantsov, Guan, & Wood, 2009). Exposure to osmotic shock can cause an immediate water efflux and changes in cell turgor that may result in dehydration and eventually in cell death. The adaptability to high osmotic stress was proposed to be in part due to accumulation of compounds such as glycine‐betaine, proline, and glutamine in the cytoplasm which can act as osmo‐protectants (Anderson & Witter, 1982; Kramer, 2010; Townsend & Wilkinson, 1992). Bacterial survival mechanisms would also involve metabolic, genomic, proteomic and structural adjustments within the cell to withstand undesirable conditions such as high osmotic pressure and low nutrient availability. It has been suggested that a suite of responses in bacteria form the basis of the heterogeneity of phenotypes observed within the bacterial population maximizing the chances of survival (Abu‐Qatouseh et al., 2010; Alreshidi et al., 2013; Crompton et al., 2014; Morikawa et al., 2010; Onyango et al., 2012; Onyango, Dunstan, Roberts, Macdonald, & Gottfries, 2013; von Eiff, 2008).

It has recently been shown that the osmotic pressure, variations in pH and cold stress during growth can induce different phenotypes with obvious alterations in the cell wall structure of these phenotypes (Onyango et al., 2012, 2013). Fourier Transform Infrared Spectroscopy (FTIR) study has also shown that the exposure of *S. aureus* to undesirable conditions included NaCl led to a significant phenotypic shifting (Wehrli et al., 2014). Proteomic and metabolomic analysis revealed that *S. aureus* substantially altered its ribosomal proteins following growth in a broth medium in ranges of temperature, pH, and osmotic pressure that were similar to those observed in wound site conditions (Alreshidi et al., 2016). These investigations concluded that changes in the metabolome and proteome could possibly lead to significant changes in the cell size and cell wall structure resulting in phenotypic shifts such as the formation of small colony variants (SCVs). SCVs are a subpopulation of bacteria that exhibit atypical metabolism, heightened tolerance to stress and have been involved in many recalcitrant infections (Dhar & McKinney, 2007; Onyango & Alreshidi, 2018; Onyango et al., 2008). These SCV phenotypes have shown an intrinsic ability to persist in the presence of challenges including antibiotics that go beyond the classical mechanisms of antibiotic resistance (Baumert et al., 2002). SCVs have also been isolated under various environmental and laboratory conditions and exhibit a variety of morphological, ultrastructural and biochemical abnormalities in comparison to their normal type. Metabolically, some SCVs have been described as auxotrophs of haemin, menadione and thymidine (Bui, Turnidge, & Kidd, 2015; Moisan et al., 2006; Proctor et al., 2014; von Eiff, Peters, & Becker, 2006). As the proteome and metabolome constitute the structural and functional operations of living organisms, proteomic and metabolomic studies form a basis of understanding the cellular responses that provide adaptability for the bacterium under changing environmental conditions (Hecker, Becher, Fuchs, & Engelmann, 2010; Kriegeskorte et al., 2011; Liebeke et al., 2011). The studies to date have demonstrated that exposure to higher concentrations of NaCl during growth in culture media resulted in cells at mid‐exponential growth displaying alterations in cytoplasmic composition, protein composition and cell morphology compared with control cells under optimal conditions (Alreshidi et al., 2015, 2016; Crompton et al., 2014; Onyango et al., 2012). The current study addressed the next logical question as to whether or not *S. aureus* could alter the cytoplasmic amino acid metabolites and proteome under exposure conditions that do not support cell replication for population growth i.e.—can the cells adjust? The bacterium must survive harsh and variable conditions between hosts where nutrients would be limiting and survival would depend on its metabolic response capability. This study investigated the metabolic impacts on cells following incubation of washed cells in a defined minimal medium that provided an energy source (glucose) and a nitrogen source (glycine) but without any other growth factors. The incubation medium would not facilitate cell replication but would support ongoing oxidative metabolism and limited protein synthesis. It was proposed that under these conditions the cells exposed to 5% NaCl would show altered amino acid metabolites and proteome profiles within 90 min compared with controls.

## 2 MATERIALS & METHODS

### 2.1 Bacterial growth and incubation conditions

The *S. aureus* strain used in the present investigation was isolated from patients who were suffering from chronic muscle pain (Butt et al., 1998). This isolate had been used in subsequent studies to examine metabolic and proteomic acclimation to changes in the environmental conditions (Alreshidi et al., 2015, 2016; Onyango et al., 2012, 2013). The bacterial strain was grown as culture stock on horse blood agar (HBA) and preserved on sterile glass beads at −80ºC with a regular subculturing to maintain viability. The identity of the isolate was checked regularly using API™ Staph biochemistry and through the amplification of 16S rRNA gene by polymerase chain reaction (PCR) (Brown, Martin, Roberts, & Aitken, 2001).

Overnight cultures of *S. aureus* were grown in Tryptic Soy Broth (TSB) at 37°C with constant agitation (120 rpm). Eight flasks containing 95 ml TSB culture media were inoculated with 5 ml of overnight culture in 500 ml conical flasks which were incubated at 37°C with constant agitation (120 rpm) for 3 hr. Replicate cultures (*n* = 4 per treatment) were harvested and washed three times using phosphate buffer saline (PBS) to ensure the removal of TSB medium. Washed cells were then incubated in a defined minimal medium consisting of PBS with trace elements (MgSO_4_·H_2_O, 1.57 μg/ml; H_3_BO_3_, 2.8 μg/ml; Cu(NO_3_)_2_·3H_2_O, 0.04 μg/ml; ZnSO_4_·7H_2_O, 0.24 μg/ml; NaMoO_4_·2H_2_O 0.75 μg/ml), salt elements (MgSO_4_·7H_2_O 200 μg/ml; FeSO_4_·7H_2_O, 0.25 μg/ml; CaCl_2_·2H_2_O, 0.2 μg/ml), glycine (150 μg/ml) and glucose (300 μg/ml), with or without additional 5% NaCl for 90 min. This defined minimal medium did not support growth but did provide trace elements and nutrient sources of energy and nitrogen that assisted the bacterium to maintain active metabolism. Cell numbers were checked at time 0 and at the end of 90 min by spread plate methods to test for growth in cell numbers. After 90 min incubation, the cells were centrifuged at 6,000*×g* for 25 min and the supernatants were filtered through a 0.22 µm filter to facilitate analyses of glycine and glucose uptake.

### 2.2 Analyses of incubation supernatants and cell extracts for metabolic profiling

The supernatant (100 µl) of each replicate and the initial media samples were prepared for analysis by gas chromatography with a flame ionization detector (GC‐FID) to investigate glycine uptake. A second set of corresponding medium supernatant samples (200 µl) taken before and after incubation were dried and reacted to form the trimethylsilyl/methoxyamine derivatives for analysis by GC‐MS to evaluate the uptake of glucose during the incubation period as previously described (Alreshidi et al., 2015). The washed bacterial cells were immediately quenched using liquid nitrogen and subjected to lyophilization for amino acid metabolites and proteomic analyses.

Approximately 10–12 mg of lyophilized cells were suspended with 10 ml of 1:1 (v/v) of ice‐cold methanol/water. The methanol/water cell suspension was then frozen in liquid nitrogen and stored at −20°C for 30 min. Metabolites were separated from the cell debris by spinning at 6,500×*g* for 25 min. The supernatant containing the metabolites was dried using a centrifugal vacuum drier (Labconco CentriVap) followed by resuspension in 500 µl of sterile Milli‐Q water and amino acid metabolites were then analyzed by using Phenomenex^®^ EZ: faast™ analytical kit as per manufacturer's instructions. The derivatized amino acids were analyzed using a Hewlett Packard HP 6890 series gas chromatograph coupled with a flame ionization detector with Agilent Technologies Chemstation Software (Rev C.01.04(35), 2012). The injected sample volume was 2 µl with splitless mode and the flow rate of the carrier gas (Helium) was 0.5 ml/min. Norvaline was used as an internal standard to determine the concentrations of amino acids of the samples as nmol/mg cell dry weight.

### 2.3 Processing and analysis of metabolic profile data

Four replicates of the reference control and the cells exposed to osmotic stress were used in this experiment to investigate the responses of the cytoplasmic metabolites and proteome. The data generated from GC‐FID were analyzed via ANOVA to identify the amino acids that were significantly changed on exposure to osmotic stress (Statistica, TIBCO Software Inc. [2017], data analysis software system), version 13. http://statistica.io). Principal Component Analysis (PCA) was then performed employing SIMCA‐p+ (12.0, Umetrics Sweden; Alreshidi et al., 2015; Onyango et al., 2012). The data were subjected to mean centering and unit variance scaling before PCA calculations. The model complexity and validity were evaluated by cross validation as implemented in the software. The PCA figures were generated using Statistica software.

### 2.4 Proteome analysis

#### 2.4.1 Protein extraction

Protein extraction was performed as described previously (Alreshidi et al., 2015). Briefly, protein was extracted from dried cells of both unstressed and NaCl stressed samples. The cells were suspended in 500 µl of SDS Lysis Buffer [2% SDS, 0.375 M Tris (pH 6.8), 3.4 M sucrose (Sigma‐Aldrich)] with a protease inhibitor tablet (cOmplete^TM^, Mini, Roche Diagnostics) and the suspension was mixed and boiled at 100°C for 5–6 min. The supernatant containing the extracted proteins was collected and quantified by BCA^TM^ assay (Bio‐Rad) as per manufacturer's instructions, with bovine serum albumin (BSA) as the reference standard.

#### 2.4.2 Protein digestion and LC/ESI‐MS/MS analysis

Protein digestion was conducted as described previously (Alreshidi et al., 2015). Briefly, 150 µg protein from each replicate were digested by adding trypsin (Promega) in a ratio of 50:1 (protein:trypsin) in 25 mm ammonium bicarbonate and incubated overnight at 37°C with constant shaking. Reaction of trypsin digestion was stopped by addition of formic acid (1% v/v) followed by centrifugation at 23,400 x *g* for 40 min just prior to proteomic analysis.

Tryptic peptides were analyzed using liquid chromatography coupled with electrospray ionization tandem mass spectrometry (LC/ESI‐MS/MS) as described previously (Alreshidi et al., 2015). Peptide separation was performed via nanoscale reverse phase high‐performance liquid chromatography (RP‐HPLC; Dionex Ultimate 3000 RSLC nano LC System) coupled with online electrospray ionization (ESI). The samples loaded at 5 l/min for 7 min onto a 5 m C18 trap column (Acclaim PepMap100 100 m × 2 cm, nanoViper, Thermo Dionex) to wash the samples before running on analysis column (Acclaim PepMap 75 m × 15 cm, nanoViper, Thermo Dionex). Peptides were then separated using a gradient of 2%–40% solvent B (0.1% of formic acid and 80% Acetonitrile in Water), for 120 min with a flow rate of 400 nl/min. The temperature of the column was set to 35°C, and peptides eluted from the column were directly applied to the nanoflow Electro Spray Ionization source of a Bruker AmaZon ETD, 3D‐Ion Trap Mass Spectrometer (BrukerDaltonics Bremen, Germany). The MS system was set to perform a full MS scan (400–2,000 m/z) followed by MS/MS on the top 6 most intense Ions, with an exclusion time of 30 s applied.

### 2.5 Protein identification and quantification

Protein identification and quantification were performed as described previously (Alreshidi et al., 2015). Briefly, Bruker raw format (.baf) files were converted into mascot generic format using Data Analysis 4.1 (Bruker Daltonics Bremen, Germany and then imported into Bruker's Proteinscape platform and searched against the UniProt database (538,010 sequences, February, 2013) Firmicutes taxonomy (Gram‐positive bacteria) using Mascot 2.3.02 (Matrix Science) search algorithm. AmaZon ETD raw format files were converted into MZXML format using Compassxport (Bruker Daltonics, Bremen, Germany) to be imported to the commercial, label‐free quantitation, software Decyder™ MS 2.0 PepDetect module. (GE Healthcare, UK) Peptide detection and subsequent relative quantitation were then performed between control and NaCl stressed samples. Peptides in each of the reference control and NaCl stressed replicate LC‐MS runs were detected and cross‐matched according to retention time and mass to charge ratio (m/z) using the Decyder PepDetect and PepMatch modules respectively. Background subtraction modeling was set to smooth surface to model local variations in background intensity to account for varying background due to the acetonitrile gradient. Charge state assignment was set to always require a charge state assignment with three isotopic peaks required. LC peak shape tolerance, mass to charge shift and shape tolerance were set to 20%, 0.1 µ, and 5%, respectively. The intensity of detected peptides was calculated in software by integrating the area of the peptides' Extracted Ion Chromatogram and converted into a log2 value. Processed intensity maps were then submitted to the Decyder PepMatch module. Each analysis was assigned as either a control or NaCl stressed experimental group and time aligned according to Base‐peak Ion Chromatogram. Time aligned intensity maps were matched and cross detected according to retention time and m/z tolerances (within 1 min and 0.5 Da, respectively). PepMatched data were assessed manually for accuracy and normalized in software by measured intensity distribution (assumes that a majority of peptides between replicates do not vary in intensity). Peptides with a *t* test probability of <0.05 were manually validated by comparing extracted ion chromatograms (EIC) from the raw data. The matched table generated by Decyder™ MS contained 3,291 peptides which ultimately represented all detected proteins were exported to an Excel^®^ (Microsoft^®^) spreadsheet file for further data analysis using PCA (SIMCA‐p+ 12.0, Umetrics Sweden).

## 3 RESULTS

The cell numbers were determined by the plating method to assess colony forming units at time 0 for both control and NaCl stressed samples which yielded values of 5.5 ± 0.6 × 10^8^ and 5.8 ± 0.8 × 10^8^ (±*SD*), respectively; at 90 min the cell numbers for control and NaCl stressed samples were 5.3 ± 0.5 × 10^8^ and 5.6 ± 0.9 × 10^8^ (±*SD*), respectively. These results indicated that no significant changes in population densities occurred over the incubation period. Initially, the control and 5% NaCl treatment incubation media each contained 730 nmoles/ml of glycine; after 90 min incubation at 37°C, 147 and 103 nmoles/ml, respectively, remained in the media supernatants. The NaCl treated cells used significantly more glycine than the controls. The initial glucose concentrations were 1,665 nmoles/ml and were almost completely utilized in both treatments; 3.9 nmoles/ml remained in the supernatant from the controls and 0.7 nmoles/ml remained in the supernatant from the 5% NaCl treatments.

Analyses of the cytoplasmic extracts identified 13 amino acids and one dipeptide in both treatment and control samples. Glycine, aspartic acid, and glutamic acid collectively comprised 87% and 89% of the cytoplasmic amino acids, respectively, in control and 5% NaCl treatments. The total level of cytoplasmic amino acids in the 5% NaCl treatment (239 nmol/mg dry biomass) was significantly higher than the corresponding control (206 nmol/mg dry biomass). The increase was primarily attributable to the higher levels of proline and glutamic acid but increases were also noted for serine, asparagine, and ornithine (Table 1). Conversely, alanine, phenylalanine, and methionine were significantly reduced in the 5% NaCl treated samples. Glycine, valine, aspartic acid, histidine, and tyrosine as well as the dipeptide glycine‐proline did not show any significant variation in their concentrations following exposure to additional NaCl.

**Table 1:** The concentrations of cytoplasmic amino acids extracted from cells incubated for 90 min in a defined medium (reference control) compared with equivalent sets of cells which were incubated in the defined medium supplemented with additional 5% NaCl. Amino acid concentrations were expressed as nmoles/mg dry cell mass (mean ± *SD*)

Amino acids	Control *n* = 4 nmoles/mg dry cell mass	5% NaCl treatment *n* = 4 nmoles/mg dry cell mass
Amino acids significantly increased		
Proline	1.6 ± 0.1	6.6 ± 0.4*
Glutamic acid	35 ± 3.02	64 ± 3.8*
Serine	1.1 ± 0.04	1.5 ± 0.09*
Asparagine	0 ± 0	0.7 ± 0.05*
Ornithine	0.7 ± 0.04	0.9 ± 0.05*
Amino acids significantly decreased		
Alanine	13 ± 0.5	10 ± 0.3*
Methionine	4.3 ± 0.4	1.9 ± 0.11*
Phenylalanine	0.4 ± 0.02	0.3 ± 0.02*
Amino acids unaltered in response to NaCl (5%)		
Glycine	94 ± 4.0	98 ± 3.2
Valine	1.8 ± 1.3	1.4 ± 0.8
Aspartic acid	51 ± 3.7	51 ± 1.7
Histidine	3.0 ± 1.4	2.4 ± 1.7
Tyrosine	0.1 ± 0.2	0.2 ± 0.05
Glycine‐proline (dipeptide)	0.5 ± 0.04	0.6 ± 0.11
Total amino acids measured for control and 5% NaCl treatment	206 ± 8.4	239 ± 7*

* *p *˂ 0.05.

**Figure 1:**
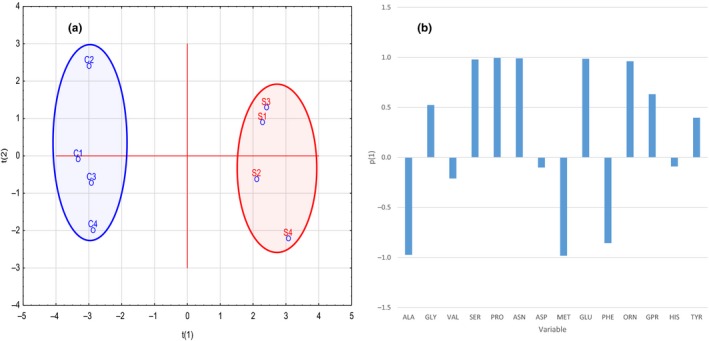
(a) Principal component analysis (PCA) scores (*t*_1_ vs. *t*_2_) plotted from *Staphylococcus aureus* amino acid data. The *S. aureus* cells harvested at mid‐exponential phase were incubated without the presence of sodium chloride representing the reference control samples (C) or incubated with the presence of additional 5% NaCl (S) before extraction of metabolites. (b) Factor coordinates of the variables generated by principal component analysis (PCA) from *S. aureus* profiles of cytoplasmic amino acid data

Principal component analysis was used to further study the difference between the profiles of cytoplasmic compositions of amino acids in the control and osmotic stressed samples. The scores obtained by PCA were plotted for each replicate which resulted in two clusters where the controls and 5% NaCl treatments were well separated by *t*_1_ scores as shown in Figure 1a. The data analysis indicated that there were two significant PCA factors according to the cross validation (CV). The PCA loadings demonstrated the contributions of the key differential amino acids to the model (Figure 1b).

**Figure 2:**
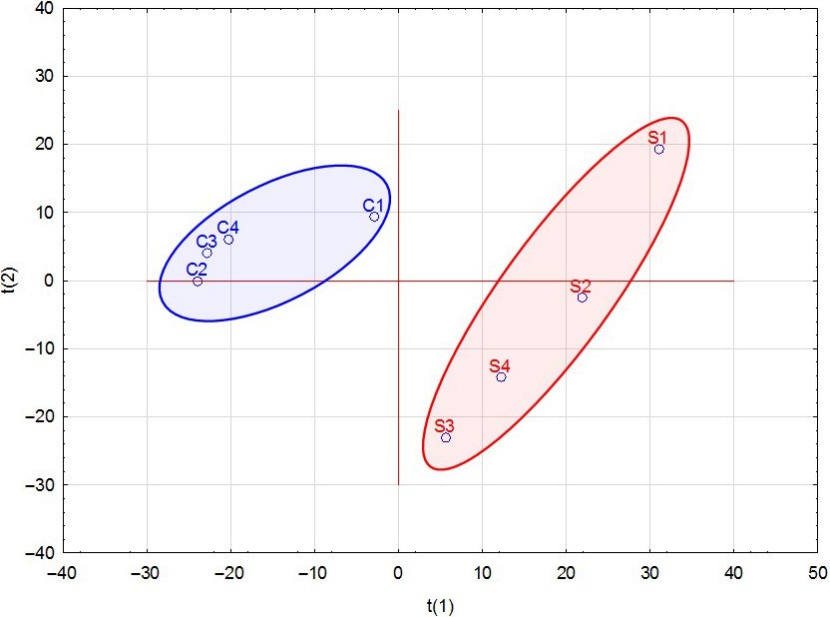
Principal component analysis (PCA) scores (*t*_1_ vs. *t*_2_) plotted from *Staphylococcus aureus* proteomic peptide data. The *S. aureus* cells were incubated in a defined minimal medium (control) or with the presence of additional 5% NaCl before extraction of proteins and analyses by LC‐MS/MS

The proteomic analyses of cytoplasmic extracts revealed that only 10 proteins had undergone statistically significant alterations in composition relative to the reference controls following the short time frame of exposure to the 5% NaCl treatment in the incubation medium which did not support growth (Table 2). Only one peptide for each protein reported was found significantly up‐ or down‐regulated in all replicates following exposure to NaCl (*p* < 0.05). However, some of the proteins had more than one peptide significantly altered but not in all replicates, so these peptides have not been included as they did not present in all samples. The low number of quantified peptides could be due to the fact that not many peptides were identified for many of the proteins (misidentification), and also could be due to the limitation of label‐free quantification. Peptide sequences and relative statistics information have been provided as supplementary data. The altered proteins were primarily associated with processes of protein biosynthesis, pathogenicity, and cell adhesion. For example, ribosomal proteins L1, L30, and S7, were found to be significantly down‐regulated in response to exposure to the additional 5% NaCl, but glycyl‐tRNA synthetase was up‐regulated. Immuno‐dominant staphylococcal antigens A and B which are associated with pathogenicity were substantially decreased in cells exposed to the 5% NaCl treatment. Pyruvate kinase was down‐regulated, whereas NADH dehydrogenase‐like protein was up‐regulated in the cells incubated with 5% NaCl. PCA for the complete proteomic dataset was carried out to further investigate proteome profiles rendering a two‐component model as validated by cross validation (CV). The PCA scores for each replicate revealed good separation between the two groups along the *t*_1_ axis, indicating differential proteomes following exposure to 5% NaCl (Figure 2).

**Table 2:** Summary of altered proteins in response to the 5% NaCl treatment

Biological process	Protein name	Accession	Peptide sequences	*t* test *p*	Av. Diff. (2Log)	Regulation	Mascot score	MW kDa
Protein biosynthesis	30S ribosomal protein S7	RS7_STAAB	R.WLVNYAR.L	<0.001	2.82	Down‐regulated	30.08	17.8
	50S ribosomal protein L1	RL1_STAAB	K.KVSISTTMGAGVAVDQASLNTQA.	<0.001	1.63	Down‐regulated	43.3	24.7
	50S ribosomal protein L30	RL30_STAS1	K.TNSSVVVEDNPAIR.G	<0.001	4.02	Down‐regulated	72.76	6.5
	Glycyl‐tRNA synthetase	SYG_STAAC	K.IFEQLSSK.F	<0.001	−0.16	Up‐regulated	39.9	53.6
Pathogenicity	Immuno‐dominant staphylococcal antigen B precursor	ISAB_STAAB	K.GNEASQLQFVVK.*N*	<0.001	3.46	Down‐regulated	55.99	19.3
	Immuno‐dominant staphylococcal antigen A precursor	ISAA_STAAC	R.LSNGNTAGATGSSAAQIMAQR.T	<0.001	4.32	Down‐regulated	98.88	24.3
Cell adhesion	Serine‐aspartate repeat‐containing protein D	SDRD_STAAE	K.FQYTNSESPTLVQMATLSSTGNK.S	<0.001	0.45	Down‐regulated	48.09	142.7
	Uncharacterized lipoprotein	Y2315_STAA3	K.SSYVAPYYGQNAAPVAR.Q	<0.001	3.03	Down‐regulated	31.9	23.3
Carbohydrate metabolism and energy production	Pyruvate kinase	KPYK_STAAC	K.ALGLITEENGITSPSAIVGLEK.G	<0.001	0.71	Down‐regulated	47.07	63.1
	NADH dehydrogenase‐like protein	Y802_STAAN	K.VLVLGAGYAGLQTVTK.L	<0.001	−1.46	Up‐regulated	75.64	44.1

## 4 DISCUSSION

*Staphylococcus aureus* cells responded to the osmotic challenge under limited growth conditions by displaying substantial alterations in amino acid and protein composition in the cytoplasm. Alterations in metabolic homeostasis may have resulted from a selection process, whereby exposure to the higher levels of NaCl selected for the proliferation of more favorable phenotypes originally present in the starter population of washed cells (Abu‐Qatouseh et al., 2010; Morikawa et al., 2010; von Eiff, 2008). However, there was no evidence of cell proliferation observed in the control cultures and nor was there any evidence of a decline of cell numbers in the NaCl treatments. These data therefore supported the hypothesis that the cells could undergo significant changes in metabolic homeostasis in response to the challenge of an additional 5% NaCl without undergoing cell replication. NaCl treated cells utilized more glycine from the medium than the controls. This could potentially reflect a higher use of nitrogen substrates to support the response to exposure to additional NaCl as observed previously under conditions of osmotic stress (Vilhelmsson & Miller, 2002). It was noted, however, that both controls and NaCl treated cells had similar levels of cytoplasmic glycine after 90 min of incubation. Glycine is normally present in concentrations around 1–2 nmoles/mg dry cell mass at mid‐exponential growth in broth culture (Alreshidi et al., 2016) but appears as the major cytoplasmic component during incubation in the minimal medium with glycine as the sole nitrogen substrate. Increased levels of glutamic acid and proline were the major responses observed in this study. It has been previously suggested that increased concentrations of cytoplasmic proline may provide a role in osmo‐protection (Anderson & Witter, 1982; Kramer, 2010; Townsend & Wilkinson, 1992). Increases in cytoplasmic proline can lead to destabilizing the DNA double helix by reducing its melting temperature which can minimize the impacts of salts on DNA (Sreedharan, Oram, Jensen, Peterson, & Fisher, 1990). It has also been shown that proline plays an important role in protein solubilization (Rajendrakumar, Suryanarayana, & Reddy, 1997; Shebuski, Vilhelmsson, & Miller, 2000). A similar increase in glutamic acid was observed in *Bacillus subtilis* following exposure to 400 mM NaCl (Whatmore, Chudek, & Reed, 1990) noting that glutamic acid was the precursor for the synthesis of proline (Kanehisa & Goto, 2000). Glutamic acid can readily be used for energy production by oxidation and it is required for the synthesis of the peptidoglycan bacterial cell wall, especially relevant if cell wall thickening is part of the response to a NaCl challenge (Doublet, Heijenoort, & Mengin‐Lecreulx, 1992). It has been shown that in staphylococcal species, the thickness of the cell wall was significantly altered following exposures to environmental changes including elevated NaCl (Onyango et al., 2012, 2013).

Reductions in cytoplasmic amino acids have been previously attributed to slower rates of metabolism to conserve energy during stressful conditions (Balaban, Merrin, Chait, Kowalik, & Leibler, 2004; Duval, Mathew, Satola, & Shafer, 2010). However, under conditions of additional 5% NaCl in a minimal medium, the only reductions observed in amino acid composition were for alanine, methionine and phenylalanine. It was proposed that these three amino acids were utilized in the response to the osmotic challenge at a faster rate than the other amino acids, and/or their rate of replenishment by synthesis may have been specifically limited by the additional NaCl in the minimal medium. There were no changes in response to the additional NaCl exposure in the abundances of the predominant amino acids, glycine and aspartic acid, in the *S. aureus* cytoplasm. The cells displayed comparable rates of glucose uptake, but significantly more glycine was taken up by the NaCl treated cells. The corresponding lack of increased glycine levels in the cytoplasm of the treated cells, despite increased uptake, suggested that there was a higher rate of usage of glycine by the cells.

The changes observed in the metabolome and proteome may represent a phenotypic shift that can occur within an existing population of cells without cell division. Such a shift may be indicative, for example, of the formation of small colony variants that have been previously shown to form in response exposures to NaCl, with accompanying reduced metabolism compared to wild‐type bacteria (Crompton et al., 2014; Onyango et al., 2013; Proctor et al., 2014; von Eiff, 2008). Prior investigations have shown that small colony variants have slower growth rates, reduced production of virulence factors and some are auxotrophic for important compounds such as haemin and menadione (Bui et al., 2015; Moisan et al., 2006; Proctor et al., 2014; von Eiff et al., 2006). On this basis, a hypothesis was suggested whereby the bacteria are continually sensing and responding to the changes in the environmental conditions to induce the most effective and efficient phenotype for survival in undesirable conditions (Casadevall, 2008; Crompton et al., 2014; de Jonge, Chang, Gage, & Tomasz, 1992; Onyango et al., 2012). It is also possible that this would be associated with the bet‐hedging phenomena where different phenotypes exist within a population with the most predominant phenotype in the population determined by the surrounding conditions (Kussell & Leibler, 2005; Kussell, Kishony, Balaban, & Leibler, 2005). However, the results of this study support the hypothesis that cells can change or adapt homeostasis via an epigenetic response.

Proteomic analysis revealed that NADH dehydrogenase‐like protein was up‐regulated in the cells incubated with 5% NaCl. In a similar manner, this protein was up‐regulated in clinically derived small colony variants of *S. aureus* that were isolated from a patient who was extensively treated with the antibiotic gentamicin (Kriegeskorte et al., 2011). The gene encoding NADH dehydrogenase was found to be significantly up‐regulated following the exposure to low pH (Bore, Langsrud, Langsrud, Rode, & Holck, 2007). Glycyl‐tRNA synthetase was also up‐regulated in the NaCl treated cells and may reflect a requirement of glycine for building extra cell wall peptidoglycan for *S. aureus*. Previous studies have shown that increased levels of cell wall peptidoglycan biosynthesis occur in *S. aureus* in response to exposure to certain stressors (de Jonge et al., 1992; Onyango et al., 2012; Sianglum, Srimanote, Wonglumsom, Kittiniyom, & Voravuthikunchai, 2011). The gene of Glycyl‐tRNA synthetase has also been found to be highly expressed in biofilm cells in comparison to planktonic cells (Beenken et al., 2004). This might suggest that this protein plays an important role in survival under certain stressful conditions.

However, several proteins such as the pathogenicity‐associated proteins, immuno‐dominant staphylococcal antigen A and B precursors and serine‐aspartate repeat‐containing protein D, were notably reduced in NaCl treated cells. This decrease suggested that *S. aureus* elicited a response by down regulating proteins associated with cell adhesion and pathogenicity with a view to minimizing the metabolic rate, preserving cellular integrity and being ready to reactivate rapidly if an appropriate opportunity was presented (Antti et al., 2013; Proctor et al., 1998; Tuchscherr et al., 2011). Four proteins known to be involved in the process of protein biosynthesis were also reduced. Reduction in these proteins further supports the interpretation that *S. aureus* was adjusting the cellular homeostasis under exposure to the additional loads of NaCl (Keren, Shah, Spoering, Kaldalu, & Lewis, 2004; Lewis, 2010). The ultimate endpoint of homeostasis measured after 90 min of exposure to 5% NaCl treatment appeared to reflect a different phenotype with altered amino acid metabolites, proteome, and pathogenic capacity. The similar rates of glucose uptake and higher levels of glycine uptake suggested that rates of nutrient utilization to support metabolism were not impaired in the presence of the additional NaCl. Under these conditions, it was proposed that the energy and organic resources were re‐purposed to facilitate the phenotypic shift and production of osmo‐protectant components. These carbohydrate and nitrogen substrates could also be converted to storage products in preparation for fueling a reverse shift to pathogenic mode when the opportunity next presented.

## 5 CONCLUSION

*Staphylococcus aureus* showed an adaptive response to exposure to a 5% NaCl treatment under conditions which limited growth and cell replication. After 90 min, the cellular responses involved alterations in the amino acid metabolites with specific increases noted for the cytoplasmic concentrations of proline and glutamic acid in the NaCl treated cells. Shifts in the proteome indicated that the NaCl treated cells had down‐regulated proteins that were consistent with a reduced pathogenic capability and lower metabolic rates. It was proposed that the alterations in metabolites and proteins formed the basis for the adaptive response required to facilitate survival under these conditions.

## CONFLICT OF INTEREST

The authors have declared that no conflict of interests exists.

## AUTHORS CONTRIBUTION

MA, RHD, JG, MMM, and TKR conceived and designed the experiments. MA, NDS, and RHD performed the experiments. MA, RHD, JG, and NDS analyzed the data. MA and RHD wrote the manuscript with support from all authors.

## ETHICS STATEMENT

None required.

## DATA ACCESSIBILITY

All data are provided in full in the results section as well as in the supplementary material.
